# Soilless biofortification, bioaccessibility, and bioavailability: Signposts on the path to personalized nutrition

**DOI:** 10.3389/fnut.2022.966018

**Published:** 2022-10-04

**Authors:** Massimiliano Renna, Massimiliano D’Imperio, Stefania Maggi, Francesco Serio

**Affiliations:** ^1^Department of Soil and Food Science, University of Bari Aldo Moro, Bari, Italy; ^2^Institute of Sciences of Food Production, National Research Council of Italy, Bari, Italy; ^3^Neuroscience Institute, National Research Council of Italy, Padua, Italy

**Keywords:** *in vitro* digestion model, bioavailability, bone health, impaired kidney function, mental illnesses, modulated nutrition

## Abstract

Propelled by an ever-growing awareness about the importance of following dietary recommendations meeting specific biological requirements linked to a person health status, interest in personalized nutrition is on the rise. Soilless biofortification of vegetables has opened the door to the potential for adapting vegetable production to specific dietary requirements. The evolution of vegetables biofortification toward tailored food is examined focusing on some specific categories of people in a context of personalized nutrition instead to simple describe developments in vegetables biofortification with reference to the single element or compound not adequately present in the daily diet. The concepts of bioavailability and bioaccessibility as a useful support tool for the precision biofortification were detailed. Key prospects for challenges ahead aiming to combine product quality and sustainable are also highlighted. Hydroponically cultivation of vegetables with low potassium content may be effective to obtain tailored leafy and fruit vegetable products for people with impaired kidney function. Simultaneous biofortification of calcium, silicon, and boron in the same vegetable to obtain vegetable products useful for bone health deserve further attention. The right dosage of the lithium in the nutrient solution appears essential to obtain tailored vegetables able to positively influence mental health in groups of people susceptible to mental illness. Modulate nitrogen fertilization may reduce or enhance nitrate in vegetables to obtain tailored products, respectively, for children and athletes. Future research are needed to produce nickel-free vegetable products for individuals sensitized to nickel. The multidisciplinary approach toward tailored foods is a winning one and must increasingly include a synergy between agronomic, biological, and medical skills.

## Introduction

According to some investigators (1), Personalized Nutrition (PN) “tailors dietary recommendations to specific biological requirements on the basis of a person’s health status and goals.” PN could be also described as a “field that leverages human individuality to drive nutrition strategies that prevent, manage and treat disease and optimize health” (2). At the same time, the term “personalization” can be interpreted both as “individualization” and “categorization,” since the two meanings can indeed coexist. In fact, although individuals can be considered unique, some can be regarded as similar enough to make up a category (3).

Generalizable nutrition recommendations can guide public policy independently of one-on-one nutrition counseling sessions. For example, the well-known Mediterranean Diet (MD) is a dietary model characterized by a high intake of vegetables and fruits leading to a reduction in blood pressure, insulin resistance, and inflammatory markers thus contributing to healthy lifestyles and practically eliminating inadequate ones (4, 5).

Not only the MD, but many other dietary patterns for human health and wellbeing have highlighted the positive role of large quantities of fresh fruits and vegetables in the daily diet (6). Nevertheless, indicating generally to eat standard portions of fruits and vegetables results inadequate to address human individuality (2). Therefore, it is important that studies in this field identify specific dietary patterns producing the most favorable impacts on health and wellbeing in similar groups of people. For example, allergen-free diets, such as nickel-free one can be tailored for specific group of people (7, 8). Adopting these types of diets can nevertheless entail limiting or eliminating multiple food products, resulting in a decrease in the quality of life. Therefore, a diet that includes vegetables with a lower nickel content than their common counterpart could be very useful for people affected by nickel hypersensitivity, since it would avoid a decrease in the quality of life.

**FIGURE 1:**
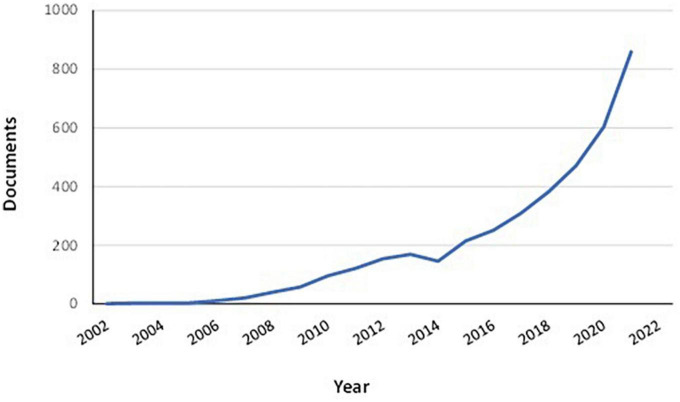
Documents regarding vegetable biofortification published from 2002 to 2021. Documents by type: article (69.3%); review (18.7%); book chapter (8.1%); conference paper (2.2%); book (1.0%); other (0.7%). Data retrieved from Scopus^®^ database (on 15 February 2022) using “biofortification” AND “vegetables” as key search terms.

At the same time people affected by some diseases or with a health status requiring higher quantities of some nutrients with respect to standard Dietary Reference Values (DRV) need to follow specific dietary guidelines. For example, the malabsorption of folic acid is a common complication of celiac disease (9). Thus, the average daily intake of folate in celiac patients is often lower with respect to that in the general population (10). Therefore, eating vegetables high in folate content could help to supplement the diet and improve the health of celiac patients. Biofortification is usually defined as the practice of deliberately increasing or decreasing the content of an essential micronutrient (i.e., vitamins and minerals) in plants (11, 12). Independently of definitions, the primary aim of biofortification is to improve the nutritional quality of fruits and vegetables for a healthier diet. Biofortification differs from conventional fortification since it is applied to crops during their growth phase. Fortification, instead, refers to the practice of adding micronutrients to food products during the processing phase of food production (13). Biofortification is a process that can be applied to fresh, uncooked vegetables. Increasing research activities have been directed toward the biofortification of vegetables in the last few years (Figure 1). Moreover, some mineral-enriched vegetables are already present on the market (14, 15). But although biofortified vegetables are becoming more and more popular, there are only limited data regarding their potential role in personalized nutrition. In the light of these considerations, this review aims to provide up-to-date information regarding biofortified vegetables for specific categories of people whose health could be enhanced by personalized nutrition plans.

## From vegetable biofortification to tailored food

Although biofortification strategies can include agronomic practices, conventional plant breeding and genetic engineering methods, the first is considered the most promising because it is the least expensive and requires only simple tools and techniques to modify the content of specific compounds in plants (16).

Agronomic practices can be considered the “starting point” of biofortification strategies in light of the fact as they were first used in the “Finland case.” In fact, since 1984 agricultural fertilizers in Finland have been supplemented with sodium selenate in an attempt to improve the nutritional quality of local foodstuffs known to be exceptionally low in selenium. This agronomic strategy affecting several crops and producing higher selenium concentrations in different food items has been proving effective since 1985, and, in fact, the selenium intake in the Finn population has increased significantly (17). We must nevertheless bear in mind that prolonged fertilization application using an enriched fertilizer may modify the soil chemical characteristics and may have a potentially negative environmental impact. It goes without saying that more agronomic practices for improving the soil’s health and the nutritional value of crops need to be identified.

**FIGURE 2:**
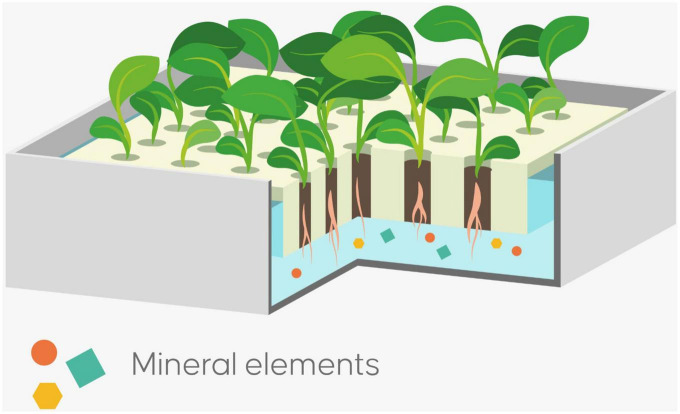
Floating hydroponic system; plants are grown in liquid culture and are fed through a nutrient solution with macro and micro-nutrient essential for plant growth.

When soilless cultivation systems are used, the soil is replaced by a substrate; plants are grown in liquid culture and are fed through a nutrient solution containing all needed elements (Figure 2). Soilless cultivation is considered an advanced, environmentally friendly agriculture practice for enhancing the quality of fresh vegetables (18).

In fact, although soilless cultivation systems have been developed primarily to address the challenge of excessive soil pathogens, it is nonetheless true that they also favor optimal control of plant growth, high productivity, and an efficient use of water and fertilizers (19). Furthermore, soilless systems represent an opportunity to modulate the nutrient solution precisely and efficaciously in the effort to improve both its organoleptic and bioactive quality traits (20). Soilless cultivation systems are being used for all of these reasons to monitor nutrient content and to enhance the quality of fresh vegetables. In fact, the constant exposure of the roots to the nutrient solution without soil interaction can maximize their uptake, translocation, and accumulation in the vegetable edible parts (20). At the same time, these novel cultivation systems, especially hydroponic ones, enhance the nutrient content of some vegetables, and thus promote the production of biofortified vegetables for personalized nutrition (21, 22).

The idea of personalized nutrition is based on the widely accepted concept of medically tailored meal programs that are prescribed to patients with specific diseases, such as diabetes (23). The idea of using some foods with specific nutritional traits for a personalized nutrition can be considered advantageous from both clinical and quality of life viewpoints (24). The soilless production of biofortified vegetables could thus represent an important strategy for directly obtaining tailored fresh vegetables without any processing steps in between.

## Biofortified vegetable products for specific categories of people

This section presents current up-to-date knowledge about the use of soilless systems to produce biofortified vegetables focusing on three categories of individuals who might benefit the most from their distinctive peculiarities: (a) patients with impaired kidney function; (b) individuals diagnosed with or at risk of osteopenia/osteoporosis; (c) persons suffering from mental illnesses.

(a) Impaired kidney function

Although potassium (K) is an essential nutrient of the human body, groups of people with reduced renal potassium excretion are sensitive to the intake of K recommended by the World Health Organization (at least 3,510 mg day^–1^ in the adult population) (25). The first group on this list is that of subjects with chronic kidney disease (CKD). Together with other so-called “lifestyle-related diseases,” the condition represents a global problem (26). It is estimated that about 10% of the worldwide population is affected by CKD, and millions die each year because they do not have access to affordable treatments. As opposed to that of the healthy population, the K intake for people affected by CKD is generally restricted to 1,500 mg day^–1^ (27) in the effort to avoid adverse effects on heart function due to disturbances in plasma potassium concentrations, commonly known as hyperkalemia (28). It is important to note that in addition to patients with CKD, individuals taking medicines such as mineralocorticoid receptor antagonists affecting the renal excretion of potassium, need a lower K intake with respect to the healthy population (28).

Since vegetables contain high concentrations of potassium (29), individuals with impaired kidney function are generally told to avoid raw vegetables and to eat only small quantities of cooked ones given that K content is reduced by leaching and boiling (30). While it is true that boiling vegetables reduces their K content, it should also be remember other important compounds for human health, such as hydrophilic vitamins, are also lost (31). Another important consideration is that people who are accustomed to frequently eating raw vegetables will almost certainly find it difficult to change their eating habits. Clearly, people with impaired kidney function would benefit if vegetables containing lower K content become available. Experimental results regarding vegetables production with low K content are summarized in Table 1.

**TABLE 1:** Reduced-potassium vegetable products for people with impaired kidney function.

Genotype	Vegetable type	Treatments	Effect	References
Chicory (*Cichorium intybus* L.)	Microgreens	Hydroponic system using polyethylene terephthalate fiber pads as growing medium and 0, 29.1, 58.4, and 117 mg of K L^–1^ in the nutrient solution.	In microgreens grown using a nutrient solution without K or with 29.1 mg of K L^–1^ the K content was between 103 and 129 mg 100 g^–1^ FW. Whereas, by using a nutrient solution with 58.4 or 117 mg of K L^–1^ the K content in microgreens was between 225 and 250 mg 100 g^–1^ FW.	(24)
Lettuce (*Lactuca sativa* L. Group *crispa*)				
Spinach (*Spinacia oleracea* L.)	Baby leaf	Floating hydroponic system using a nutrient solution with 50 and 200 mg of K L^–1^ (K_200_). The lower K concentration in the nutrient solution was used over the entire growing cycle (K_50_) or only during the 7 days before harvest (K_50–7d_).	For spinach the K content in baby leaf was of 670, 624, and 490 mg 100 g^–1^ FW, respectively, for K_200_, K_50–7d_ and K_50_. For Swiss chard the K content in baby leaf was of 459, 387, and 280 mg 100 g^–1^ FW, respectively, for K_200_, K_50–7d_ and K_50_.	(21)
Swiss chard (*Beta vulgaris* L. ssp. *vulgaris*)				
Melon (*Cucumis melo* L.)	Fruit	Three experimental trials using a hydroponic system and nutrient solutions with K content as follow: (i) 39 and 156 mg of K L^–1^; (ii) 10, 19.5, 39, 78 and 156, mg of K L^–1^; (iii) 0, 10, 19.5 and 156 mg of K L^–1^. In all trials the amount of 156 mg of K L^–1^ was the standard, while all other lower treatments were applied from anthesis to harvest.	In the first trial the K content in melon fruits was of 175 and 287 mg 100 g^–1^ FW, respectively, for low and standard K content in the nutrient solution. In the second trial the K content in melon fruits was between 250 and 360 mg 100 g^–1^ FW, passing from 10 to 156 mg of K L^–1^ in the nutrient solution. In the third trial the K content in melon fruits was between 220 and 360 mg 100 g^–1^ FW, passing from 0 to 156 mg of K L^–1^ in the nutrient solution.	(32)
Melon (*Cucumis melo* L.)	Fruit	In the first trial one melon cultivar (Panna) was hydroponically grown. All plants were fertigated with a standard nutrient solution (156 mg of K L^–1^) during first 2 weeks of growing cycle; in the following 2 weeks, applied potassium was 50, 75, 100, and 125% of required potassium, while the standard solution was still used for the control. In the second trial the same experimental protocol was applied on four cultivars: Panna, Miyabi shunjuukei, Miyabi akifuyu 412, and Miyabi soushun banshun 309.	In the first trial the K content in melon fruits was of about 195 and 410 mg 100 g^–1^ FW, respectively, for plants grown with 50% of its required potassium and the control. In the second trial the average K content in melon grown under limited K supply was of about 140 mg 100 g^–1^ FW without difference among cultivars.	(33)
Tomato (*Solanum lycopersicum* L.)	Fruit	In the first trial one tomato cultivar Cindy Sweet was hydroponically grown using a nutrient solution with 39 (low) and 156 (standard) mg of K L^–1^. For each treatment, K was entirely removed from the nutrient solution either just after anthesis of the first flower (a third of the plants) or after set of the sixth fruit in the first truss (a third of the plants). In the second trial four cultivars (Aichan, Yellow Olle, Frutica, and Cindy Sweet) were hydroponically grown using a nutrient solution with 39 (low) and 156 (standard) mg of K L^–1^. For plants treated with low-K solution, K was entirely removed from the solution after the first flower of the third truss reached anthesis.	In the first trial fruits K content was highest (202 mg 100 g^–1^ FW) in plants grown with standard nutrient solution and without K withdrawal and lowest (152 mg 100 g^–1^ FW) in plants grow with low nutrient solution and withdrawal at anthesis. In the second trial fruit K content in plants grown with standard nutrient solution was on average 242, 250, 193, and 185 mg 100 g^–1^ FW, respectively, for Aichan, Yellow Olle, Frutica, and Cindy Sweet; fruits K content in plants grown with low nutrient solution was of (on average) 95, 134, 133, and 136 mg 100 g^–1^ FW, respectively, for Aichan, Yellow Olle, Frutica, and Cindy Sweet.	(34)

FW, fresh weight.

Some investigators used a hydroponic system to produce micro chicory and micro spinach without K or with 29.1 mg of K L^–1^ (24). They reported that 100 g of low K content microgreens provided only 7.7–8.6% of the K daily intake recommended for people affected by CKD, while the same serving size of their mature counterparts provided approximately a fourfold higher K intake (24).

A hydroponic floating system of spinach and Swiss chard using a nutrient solution with 50 mg of K L^–1^ led to a significant decrease in K in baby-leaves for both (on average 27 and 39%, respectively, for spinach and Swiss chard) with respect to the K concentration (200 mg L^–1^) usually used to grow baby leaf vegetables in hydroponic conditions (21). Since both of these leafy greens are commonly considered potassium-rich vegetables, these experimental results confirm that the commercial production of baby-leaves of spinach and Swiss chard is possible.

When the K content in the nutrient solution was reduced from 156 to 0, the decrease in K in hydroponically grown melons was between 20 and 40% (32). Likewise, melon plants grown with 50% of the required K, produced fruits with a K content about 53% lower with respect to a standard solution of 156 mg of K L^–1^, excluding other differences among the cultivars (33). These results highlight that 100 g of low-K content melons would provide about 9.3–14.7% of the K daily intake recommended for people affected by CKD, while the same serving size of conventionally grown melons would provide an approximate threefold higher intake of K (32, 33).

The hydroponic production of tomatoes using a nutrient solution of 39 mg of K L^–1^ and completely removing K from the nutrient solution after anthesis led to a cultivar-dependent decrease of K (between 40 and 60%) in the tomatoes with respect to using a standard solution containing 156 mg of K L^–1^ (34). These low-K content tomatoes would provide about 6.3–9.1% of the K daily intake recommended for people affected by CKD, while the same serving size of conventionally grown tomatoes would provide approximately a threefold higher K intake (34).

Overall, these experimental results demonstrate that hydroponic production technologies to produce vegetables with a low potassium content can obtain low-K products for people with impaired kidney function. Reducing K content in fruit appears nevertheless to be a more complex process as the growing cycle is longer. A transition from the vegetative to the reproductive phase is also involved and it is difficult to decide the right time to drastically reduce or entirely remove K from the nutrient solution without negatively affecting yield and/or the quality of the vegetable products. In fact, in Japan a standard solution of 156 mg L^–1^ of K, which was used in the studies described above, is widely utilized for the hydroponic cultivation of tomatoes and melons (33, 34). In other countries, however, 150 mg L^–1^ is already considered a low K concentration for tomatoes grown hydroponically, while quantities of 300 and 450 mg of K L^–1^ are considered, respectively, as medium and high (35).

(b) Bone health and osteoporosis

The human skeletal system is a complex organ in constant equilibrium with the rest of the body. In addition to providing structural support for the body, bone is the major reservoir for many minerals and compounds essential for maintaining a healthy pH balance (36). Bone health is the resultant of bone mass, bone architecture, and body mechanics. Illnesses like osteoporosis, characterized by low bone mass and microarchitectural deterioration of the bone tissue lead to decreased bone strength and increased risk of low-energy fractures, or so-called fragility fractures (37). Bone Mineral Density (BMD) is the measure that is commonly used to quantify bone health. A lower BMD value indicates an increased risk of osteoporosis or fractures. Osteoporotic fractures are a major cause of morbidity and disability in the elderly population and, in the case of hip fractures, can lead to premature death. It is important to remember nevertheless that although the deterioration of the body during the aging process renders the older adult particularly susceptible to poor bone health, osteoporosis should not be considered a disease exclusively pertaining to elderly individuals as it globally affects millions of men and women of all ages and ethnicities (38, 39).

Bone mass is influenced by factors such as sex, hormones, as well as genetic and environmental variables and last, but not least, nutrition. From a nutritional viewpoint, it is well known that the optimal intake of calcium (Ca) and vitamins D and K are important factors in the primary as well as secondary prevention of osteoporosis (38). It is also well known that the daily recommended intake of Ca for individuals between 19 and 50 is 1,000 mg, while it is 1,200 mg for those over 50 (40). Silicon is another element that has been associated with promoting bone formation and increasing BMD in men and premenopausal women (41, 42). It would seem that an intake between 10 and 25 mg per day is able to improve bone health (43, 44). Furthermore, some data indicate that between 1.0 and 3.0 mg per day of Boron (B) has beneficial effects on bone health (43–45). It is important to remember nevertheless, that there is no single food or nutrient capable of ensuring bone health on its own. Instead, a balanced diet with appropriate quantities of fruits and vegetables containing vitamins, minerals, and alkalinizing substrates is thought to be the best approach (38, 39). In the light of these remarks, eating vegetables with a high content of Ca, Si, and B appears to be an efficacious way of promoting bone health particularly in people who are susceptible to osteoporosis. Examples of biofortified vegetables with Ca, Si, and B are reported in Table 2.

**TABLE 2:** Biofortified vegetables with calcium, silicon and boron indicated to people for whom it is desirable to promote bone health.

Element	Genotype	Vegetable type	Treatments	Effect	References
Silicon	Chicory (*Cichorium intybus* L.)	Baby leaf	Floating hydroponic system by adding 0, 50, and 100 mg of Si L^–1^ in the nutrient solution.	The added silicon in nutrient solution caused a species-related accumulation of Si: from 8 to 32 mg kg^–1^ FW in tatsoi, from 9 to 50 mg kg FW in mizuna, from 7 to 43 mg kg^–1^ FW in purslane, from 19 to 137 mg kg^–1^ FW in basil, from 8 to 36 mg kg^–1^ FW in Swiss chard, and from 11 to 36 mg kg^–1^ FW in chicory.	(12)
	Basil (*Ocimum basilicum* L.)				
	Swiss chard (*Beta vulgaris* L. ssp. *vulgaris*)				
	Purslane *(Portulaca oleracea* L.)				
	Tatsoi (*Brassica rapa* L., Tatsoi group)				
	Mizuna (*Brassica rapa* L., Mizuna group)				
Silicon	Green bean (*Phaseolus vulgaris* L.)	Fruit	Hydroponic system using perlite as growing medium and adding 0 (unbiofortified) and 100 mg of Si L^–1^ (biofortified) in the nutrient solution.	Silicon biofortification allowed to increase silicon content in pods from 8.9 (unbiofortified) to 26.0 (biofortified) mg 100 g^–1^ FW.	(47)
Silicon	Strawberry (*Fragaria* × *ananassa*)	Fruit	Hydroponic system by adding 0 (control), 50 (Si-50), and 100 (Si-100) mg of Si L^–1^ in the nutrient solution.	Silicon content in strawberry was 6.4, 30.0, and 85.0 mg 100 g^–1^ FW, respectively, in “Control,” “Si-50” and “Si-100.”	(48)
Silicon	Melon (*Cucimus melo* L.)	Fruit	Hydroponic system using a mixture of perlite-peat as growing medium and adding 0 and 100 mg of Si L^–1^ in the nutrient solution. Two Italian landraces of melon (Carosello and Barattiere) were used in the experiment.	Only for the Carosello the Si concentration in fruits increased from about 22.5 to 43.9 mg 100 g^–1^ FW, passing from 0 to 100 mg Si L^–1^ added in the nutrient solution.	(49)
Silicon	Spinach (*Spinacia oleracea* L.)	Baby leaf	Floating hydroponic system with three Si level in the nutrient solution: 2 (control), 100 (Si-100), and 200 (Si-200) mg L^–1^.	Silicon content in spinach was of 1.13, 4.38, and 4.30 mg 100 g^–1^ FW, respectively, in “Control,” “Si-100” and “Si-200.”	(50)
Silicon	Chicory (*Cichorium intybus* L.)	Baby leaf	Floating hydroponic system using a nutrient solution with four combination of added Si and NaCl levels: (i) 0 mg of Si L^–1^—0 mg NaCl L^–1^ (“Control”); (ii) 100 mg Si L^–1^ - 0 mg of NaCl L^–1^ (“Si”); (iii) 0 mg Si L^–1^–2,922 mg of NaCl L^–1^ (“NaCl”); and iv) 100 mg of Si L^–1^ Si – 2,922 mg of NaCl L^–1^ (“Si + NaCl”).	Silicon content in baby leaf was of 1.14, 1.97, 3.06 and 11.4 mg 100 g^–1^ FW, respectively, in “Control,” “NaCl,” “Si” and “Si + NaCl”	(51)
Calcium	Endive (*Cichorium endivia* L.)	Baby leaf	Floating hydroponic system using 100 (unbiofortified) and 200 (biofortified) mg of Ca L^–1^ in the nutrient solution.	Calcium biofortification (200 mg L^–1^) allowed to significantly increase Ca content in all genotypes. On average, calcium content in baby leaf increased from 109 mg 100 g^–1^ FW (unbiofortified) to 120 mg 100 g^–1^ FW (biofortified).	(52)
	Basil (*Ocimum basilicum* L.)				
	Tatsoi (*Brassica rapa* L., Tatsoi group)				
	Mizuna (*Brassica rapa* L., Mizuna group)				
Calcium	Lettuce (*Lactuca sativa* L.)	Leaf	Floating hydroponic system using a nutrient solution with six different concentrations of added calcium: 0, 100, 200, 400, 600, 800 mg of Ca L^–1^.	The highest Ca content (204 mg 100 g^–1^ FW) was found in lettuce grown by using 800 mg of Ca L^–1^ in the nutrient solution. No differences were found among all other treatments with an average Ca content in lettuce of about 35 mg 100 g^–1^ FW.	(53)
Calcium	Lettuce (*Lactuca sativa* L.)	Leaf	Floating hydroponic system using a nutrient solution with four different concentrations of added Ca: 50, 100, 150, and 300 mg L^–1^. The experiment was conducted in growth chambers set at 21°C and 28°C under a 16 h photoperiod.	Only at 28°C the Ca concentration in the lettuce leaves increased from about 175 to about 220 mg 100 g^–1^ FW, passing from 50 to 300 mg Ca L^–1^ in the nutrient solution.	(54)
Boron	Purslane *(Portulaca oleracea* L.)	Baby leaf	Floating hydroponic system using a nutrient solution with 0.3, 3, and 6 mg of B L^–1^.	In the first trial the B content in purslane was of 0.5, 3.1, and 5.1 mg 100 g^–1^ FW, respectively, for 0.3, 3, and 6 mg of B L^–1^ in the nutrient solution. In the second trial the B content in purslane was of 1.2, 2.3, and 3.4 mg 100 g^–1^ FW, respectively, for 0.3, 3, and 6 mg of B L^–1^ in the nutrient solution.	(22)

FW, fresh weight.

Silicon in the range of 50–100 mg L^–1^ in the nutrient solution was used to biofortify a series of baby-leafy vegetables (chicory, basil, purslane, Swiss chard, tatsoi, and mizuna). The biofortified vegetables showed, on average, more bioaccessible Si with respect to the unbiofortified ones (12). It was found that 100 g of these biofortified vegetables could provide approximately 13, 20, 17, 55, 14, and 14% of silicon intake (25 mg/day), respectively, for tatsoi, mizuna, purslane, basil, Swiss chard and chicory. All of these vegetables can be eaten raw or cooked; silicon-biofortified-basil can also be used to make a pesto sauce (46), a particularly healthy dish for persons with risk factors for osteoporosis.

Silicon-enriched green beans through soilless cultivation has produced a Si concentration about 192% higher than that in controls (47). Therefore, the full daily intake of Si (25 mg Si per day) could be satisfied by consuming about 96 g of biofortified green beans. Some investigators reported that the Si content of biofortified pods is higher than that in unbiofortified ones even after cooking, regardless of the cooking method used. Furthermore, Si bioaccessibility in the cooked pods was more than tripled following biofortification (47).

In a study evaluating silicon biofortification of strawberries, the Si content was increased with respect to that in controls, obtaining 3.7–12.2-fold higher values by using, respectively, 50 and 100 mg of Si L^–1^ in the nutrient solution (48). These results suggest that it is possible to satisfy the full daily intake of Si (25 mg of Si/day) by consuming 83 or 29 g of biofortified strawberries, respectively, for plants grown by adding 50 and 100 mg of Si L^–1^ in the nutrient solution.

Carosello, an Italian melon consumed ripe or immature, has been biofortified by adding 100 mg of Si L^–1^ in the nutrient solution (49). Some investigators reported identifying a Si concentration in these landraces about 95% higher than that in plants grown using a nutrient solution without added Si. These results suggest that it is possible to satisfy the full daily intake of silicon (25 mg/day) by consuming only 57 g of biofortified Carosello fruits.

Quantities higher than 100 mg of Si L^–1^ were added to the nutrient solution in a study evaluating the Si biofortification of baby leaf spinach (50). The investigators reported a Si content about 288% higher in spinach grown using 100 mg Si L^–1^ in the nutrient solution with respect to that in the controls (2 mg of Si L^–1^); no further increase in Si content in the spinach was found when addition in the nutrient solution went from 100 to 200 mg of Si L^–1^ (50). According to that study, 100 g of biofortified spinach provided 17% of the Si intake (25 mg per day), independently of the quantity of Si (100 or 200 mg L^–1^) added to the nutrient solution.

Other investigators (51) found that the biofortification of chicory plants with Si in combination with NaCl supplementation enhanced the Si tissue enrichment more with respect to Si biofortification alone. Moreover, bioaccessible Si in chicory under “Si + NaCl” treatment was found to be the highest (51). According to these results 100 g of chicory grown using a nutrient solution enriched with Si in combination with NaCl could supply about 46% of the Si intake (25 mg per day). It is important to remember that eating 100 g of silicon-biofortified chicory under salinity stress conditions would mean consuming 190 mg of Na, a value that could be considered negligible as far as the recommended limits are concerned (51).

As far as Ca is concerned, some investigators (52) found that the average Ca content increased by 9.5% in four types of baby leaves (endive, basil, tatsoi, and mizuna) when the addition in the nutrient solution went from 100 to 200 mg of Ca L^–1^. Moreover, Ca bioaccessibility ranged from 25% (basil) to 40% (endive), and the biofortified vegetables showed more bioaccessible Ca. On average, the consumption of 100 g of Ca biofortified baby leaf vegetables would provide an intake of 119 mg of Ca, equivalent to 10–12% of the daily intake.

Adding 800 mg of Ca L^–1^ to the nutrient solution led to a Ca concentration in lettuce about fivefold higher with respect to that for other treatments (53). It is interesting to note that 100 g of lettuce biofortified using 800 mg of Ca L^–1^ in the nutrient solution can supply about 20% of the Ca daily intake; the same serving size of lettuce after lower quantities of Ca biofortification supplies only 3.5% of the Ca daily intake.

Another study aiming to evaluate the increase in calcium content in lettuce leaves grown at different temperatures uncovered that the Ca concentration increased by about 26% rising from 50 to 300 mg of Ca L^–1^ in the nutrient solution, but only at 28°C (54). In fact, the same Ca concentration used at 21°C did not affect the Ca content in the lettuce (54). The results of this study indicate that 100 g of Ca biofortified lettuce can supply about 18–22% of the recommended daily intake.

When they evaluated boron-biofortification in purslane, a wild edible plant, some investigators found that the B content was increased with respect to that in the controls, obtaining 1.8–10.7-fold higher values by using, respectively, 3 and 6 mg L^–1^ of B in the nutrient solution. The average daily intake of B (2 mg) could thus be satisfied by consuming between 48 and 75 or 48 g of biofortified purslane (22).

Overall, these experimental results reviewed in the present manuscript show that the hydroponic cultivation of biofortified vegetables for Ca, Si, and B could be an effective way to obtain vegetable products promoting bone health. It should also be remembered that a serving size of less than 100 g of biofortified fruit or vegetables could satisfy the full recommended daily intake of Si, while a serving size of 100 g of leafy vegetables could supply only a part, generally between 13 and 55%. The Ca biofortification of vegetables would mean that 100 g of a vegetable could contain 20% of the recommended daily intake. As far as B biofortification is concerned, consuming a serving size of vegetables less than 100 g would satisfy its recommended daily intake.

Experimental results also demonstrated that progressively increasing amounts of a mineral elements in a nutrient solution does not necessarily correspond to the increase of this element in the edible part of vegetables. Moreover, it should also be remembered that a salt must be introduced into the nutrient solution to increase the content of a specific cation (such as Ca). As a consequence, even the content of undesirable anions, such as nitrate and chloride, may be higher in biofortified vegetables.

From a nutritional point of view, if a vegetable can be enriched with Ca, Si, and B simultaneously this would facilitate the production of bone-healthy vegetables in particular for individuals at risk of osteopenia/osteoporosis.

(c) Mental illnesses

Mood disorders, including bipolar disorder, represent an important category of mental illnesses, whose prevalence is generally increasing in developed countries. Lithium (Li) compounds seem to be among the most promising and effective drugs used to treat this disorder, in particular with regard to bipolar affective disorder (55). There has also been some evidence that Li may be useful in preventing neurodegenerative diseases such as Alzheimer’s disease (56) in treating depression, and in stabilizing moods due to its antimanic, antisocial, and prophylactic properties (57).

Lithium, a naturally occurring metal in the earth’s crust, is used in the form of carbonate (Li_2_CO_3_) as a treatment for psychiatric disorders. Between 600 and 1,200 mg Li_2_CO_3_ per day, containing 113–226 mg of elemental Li (58), are the usual doses prescribed for these types of disorders. It should in any case be remembered that there is some evidence that although lithium is not officially considered a micronutrient, a daily intake of 1,000 μg Li for a 70-kg adult (14.3 μg kg^–1^ body weight) could effectively prevent mood disorders and reduce impulsiveness and nervousness especially in subjects at risk (59). According to these studies, lithium-enriched foods could help to stabilize moods in those subjects. The main sources of Li in the diet are nuts, cereals, fish, and vegetables, but their percentages are negligible in many geographic regions (59).

In a study aiming to evaluate the Li intake in foods served to students, some Authors (60) found that the Li amount supplied daily *via* the diet was 10.7 μg, an intake that can be considered low with respect to the proposed amount of 1,000 μg Li per day. Thus, vegetables enriched with lithium could have a positive effect on the mental health of individuals susceptible to mental illness. Some vegetables biofortified with lithium are reported in Table 3.

**TABLE 3:** Lithium biofortified vegetable products indicated to people for whom it is desirable to promote mental health.

Genotype	Vegetable type	Treatments	Effect	References
Tomato (*Solanum lycopersicum* L.)	Fruit	Hydroponic system by adding 0 (control), 0.69, 6.89 e 34.47 mg of L^–1^ Li in the nutrient solution.	Lithium biofortification allowed to increase Li content in fruits from < 0.3 (control) to 21.8 (34.5 mg L^–1^) μg g^–1^ DW.	(61)
Lettuce (*Lactuca sativa* L. Group *crispa*)	Leaf	Foliar application by adding 0 (control), 10, 20, 30, and 40 mg of Li L^–1^), comparing two mineral sources of Li (lithium sulfate—Li_2_SO_4_—and lithium hydroxide—LiOH).	The Li content in leaves ranged from 61 μg 100 g^–1^ DW (control) to 3,770 (40 mg of Li_2_SO_4_ L^–1^) and 5,100 (40 mg LiOH L^–1^) μg 100 g^–1^ DW.	(63)
*Agrocybe cylidracea Hericium erinaceus*	Mushrooms	Growing media enrichment by adding 0 (control), 0.25, 0.5, 0.75, and 1.0 mM Li, comparing two Li salts (lithium chloride—LiCl—and lithium acetate—CH_3_COOLi).	The added lithium in growing media caused a species-related accumulation of Li in mushrooms: from about 0 to 2.44 mg kg^–1^ DW in *A. cylidracea* and from about 0.1 to 6.87 mg kg^–1^ DW in *H. erinaceus*.	(64)
*Ganoderma lucidum Pleurotus eryngii Pleurotus ostreatus*	Mushrooms	Growing media enrichment by adding 0 (control), 0.25, 0.5, 0.75 and 1.0 mM Li, comparing two Li salts (lithium chloride - LiCl - and lithium acetate - CH_3_COOLi).	The added lithium in growing media caused a species-related accumulation of Li in mushrooms: from about 0 to over 70 mg kg^–1^ DW in *G. lucidum*, from about 0 to 16.5 mg kg^–1^ DW in *P. ostreatus* and from about 0 to 15.1 mg kg^–1^ DW in *P. eryngii*.	(67)
*Lentinus crinitus*	Mushrooms	Growing media enrichment by adding 0 (control), 5, 10, 15, 20, 25, 30, 40, 50 or 100 mg of Li L^–1^, comparing two Li salts (lithium carbonate—Li_2_CO_3_—and lithium chloride—LiCl).	The lithium content in mushrooms ranged from 0 (control) to 267 mg kg^–1^ by using 30 mg of LiCl L^–1^ and from 0 (control) to 574 mg kg^–1^ by using 25 mg of Li_2_CO_3_ L^–1^.	(71)

DW, dry weight.

A study carried out some time ago evaluated Li concentrations in tomatoes, which was increased with respect to that in controls, obtaining a value 70-fold higher by using 34.5 mg of Li L^–1^ in the nutrient solution (61). The investigators reported a content of 21.8 μg of Li g^–1^ of dry weight, but they did not indicate the dry weight values. Thus, hypothesizing an average dry weight of 5.5 g 100 g fresh weight (62), a serving size (100 g) of lithium-biofortified tomatoes can supply about 12% of the recommended daily intake (1,000 μg Li per day), while the same serving size of unbiofortified tomatoes can supply less than 0.2% of the daily intake.

Another study aiming to evaluate the increase in Li content of lettuce grown using five different foliar spray concentrations (0—control, 10, 20, 30, and 40 mg of Li L^–1^) and to compare two mineral Li sources (lithium sulfate—Li_2_SO_4_—and lithium hydroxide—LiOH) (63) reported that the Li concentration in the lettuce leaves was directly proportional to the concentrations of this element sprayed on the leaves regardless of the chemical form used. The Li concentration, which was low in the control plants (61 μg 100 g^–1^ dry weight), resulted 84-fold higher when 40 mg of LiOH L^–1^ was used and 61-fold higher when 40 mg of Li_2_SO_4_ L^–1^ was used, although for both sources the highest Li levels caused about a 15% reduction in plant height with respect to that in the controls (63). Nevertheless, considering an average dry weight of 9.5 g 100 g, a serving size (100 g) of Li biofortified lettuce can supply up to 484 μg of Li (using 40 mg of LiOH L^–1^), which is about 50% of the Li daily intake.

Several studies have been carried out evaluating the biofortification of mushrooms with Li as a means to increase the daily intake of the mineral. Lithium chloride (LiCl) and lithium acetate (CH_3_COOLi) at concentrations of 0 (control) 0.25, 0.5, 0.75, and 1.0 mM were used to enrich the substrate in which *Agrocybe cylindracea* (known as poplar mushroom, velver pioppin or Yanagi-matsutake) and *Hericium erinaceus* (traditionally called lion’s mane, bearded tooth, satyr’s beard, bearded hedgehog or pom pom) (64) were grown. Although the authors of the study did not indicate the mushrooms’ dry weight, they reported that a concentration of 1.0 mM determinated a Li content in the mushrooms hundreds of times higher than that in the controls. One study reported an average dry weight of 9.3 g 100 g^–1^ fresh weight for *A. cylindracea* (65) and 11.4 g 100 g^–1^ fresh weight *H. erinaceus* (66). Thus, a serving size (100 g) of biofortified *A. cylindracea* and *H. erinaceus* would supply, respectively, up to 2.3 and 7.8% of the lithium daily intake (64).

Other Authors used LiCl and CH_3_COOLi at concentrations of 0 (control) 0.25, 0.5, 0.75, and 1.0 mM to enrich the cultivation substrate in which *Ganoderma lucidum* (known as Reishi mushroom), *Pleurotus eryngii* (also known as king trumpet mushroom) and *P. ostreatus* (also known as oyster mushroom, “hiratake,” “shimeji,” or “houbitak) (67). Similarly to the previous study, these Authors found an increase in Li hundreds of times higher with respect to that in the control when 1.0 mM was used; again, even in this study the authors did not report the dry weight of the mushrooms. An average dry weight of 11.8, 8.7, and 10.8 g 100 g^–1^ fresh weight, respectively, for *G. lucidum*. *P. eryngii* and *P. ostreatus* has been reported by other authors (68–70). It would seem then that a serving size (100 g) of biofortified *G. lucidum*, *P. eryngii* and P. *ostreatus* would supply, respectively, up to 83, 13.1, and 17.8% of the recommended Li daily intake (67).

By using LiCl and lithium carbonate (Li_2_CO_3_) at concentrations of 0 (control), 5, 10, 15, 20, 25, 30, 40, 50, or 100 mg L^–1^ to enrich the substrate for growing *Lentinus crinitus*, other Authors obtained biofortified mushrooms containing up to 26.7 and 57.4 mg Li 100 g^–1^ dry weight, respectively, with 30 mg of LiCl L^–1^ and 25 mg of Li_2_CO_3_ L^–1^ (71). The Li concentrations in the mushrooms were higher than those reported by the previous studies. In this case the authors did not provide data concerning the dry weight of the mushrooms and there is no data in the literature specifying the dry weight of *L. crinitus*. We can assume that the average dry weight is about 11 g 100 g^–1^ fresh weight given the weight of a mushroom of the same genus, namely *L. edodes* (also known as “shiitake” mushroom) (72). Based on these assumptions, the daily intake of lithium can be satisfied by consuming about 34 g of *L. crinitus* biofortified using lithium chloride or about 18 g of mushrooms biofortified using lithium carbonate (71).

Overall, the experimental results show that plants can be biofortified with Li by adding an enriched nutrient solution to the culture substrates or *via* foliar applications. They also demonstrate that progressively increasing quantities of Li in the nutrient solution corresponds to increasing quantities of the element in the edible part of the vegetables (61, 63, 67, 71). The appropriate dosage of Li in the nutrient solution to positively affect the mental health of people susceptible to mental illness is an important consideration. It would seem that a too low Li level in the nutrient solution translates into vegetable products which supply low daily intakes of Li per serving size. On the other hand, too high Li levels in the nutrient solution should be avoided, since it cannot be excluded that vegetable products with high concentrations may be harmful to human health.

## Bioavailability and bioaccessibility: A useful support tool for the precision biofortification

**FIGURE 3:**
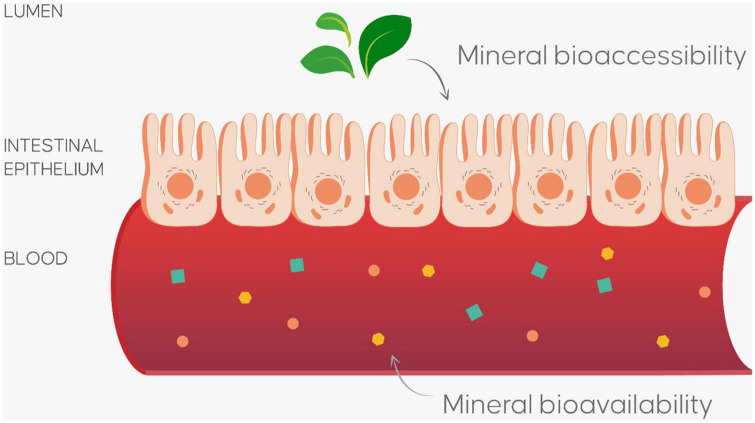
*In vitro* model for evaluate bioaccessibility (percentage of nutrient release from food matrix during gastro-intestinal digestion process) and bioavailability (percentage of nutrient adsorbed in intestinal tract after gastro-intestinal digestion process) of nutrients and/or bioactive compounds.

Whatever the biofortification approach used, obtaining biofortified vegetables represents only the first step toward achieving tailored food for personalized nutrition. The next step is determining whether the increase or reduction in a specific nutrient in the edible parts of the plant changes its bioaccessibility and bioavailability parameters. The evaluation of the benefits and/or risks associated with absorbing a particular element from biofortified vegetables must, in fact, take into consideration their bioaccessibility and bioavailability (Figure 3), which refer to the processes involved in extracting mineral elements and absorbing them; food components do not in fact exactly correspond to their functional value.

When plant foods are consumed, the nutrients (organic and inorganic) and bioactive compounds are released from the plant tissue and modified into absorbable units which can subsequently be absorbed by the epithelial cells in the gastrointestinal tract and transported to their respective target tissues (73) through the blood (74). But not all nutrients in the edible parts of a food can carry out a biological activity. Some *in vitro* models have been developed to simulate the physiological conditions of the human gastrointestinal digestion. The biochemical, physiological and dynamic conditions of the gastrointestinal system (the mouth, stomach, and intestines) such as temperature, mechanical forces, pH, the concentration of some enzymes (pepsin, pancreatin, and lipase) and the presence of bile (75) have been artificially reproduced in the gastrointestinal digestion model. The *in vitro* digestion model provides information about the amount of nutrients released from the food matrix (basically its bioaccessibility), their biochemical transformation and chemical degradation, the nutrient-nutrient and nutrient-antinutrient interactions and the effect of the matrix and food processing (75).

The release of nutrients (the bioaccessible fraction) in the intestinal tract during the digestive process of plant materials depends on a variety of factors, such as the species and the type of plant materials, the localization of the nutrient, and it is affected by a host of variables such as the concentrations of other nutrients and/or anti-nutritional compounds, the cooking method used, if and how the food has been processed, and the interaction of other nutrients. The concentration of the nutrient in the edible parts is important, but even the biofortification process can modify the release of nutrients, as has been reported by a number of studies regarding different mineral elements (12, 21, 47, 51, 52, 76).

The gastrointestinal digestion model has been used to identify the biofortified species or treatments (agronomic and/or food processing) that are able to release high quantities of silicon (12), Ca (52), zinc (76, 77), selenium (78), iodine (79), iron (80) in the intestinal tract. Thus, bioaccessibility assessment methodologies can be used to select the species, cultivars and/or genotypes that are able to release high quantities of nutrients during the digestion process in order to maximize the health effects of the biofortification process.

The bioavailability fraction can be used to evaluate bioavailability *via* an established bioassay (81); this parameter has been defined as the quantity of nutrient/s adsorbed, in the intestinal tract during the gastrointestinal digestive process (82). In general, mineral bioavailability needs to be evaluated *via in vivo* human studies. Some *in vitro* models have been proposed as an alternative to using animal models to evaluate mineral bioavailability to estimate nutritional efficiency (bioefficacy) and to determine the potential health effects of the biofortification process (79, 83–85). Currently, there are several *in vitro* models capable of simulating the intestinal mucosa with phenotypic characteristics comparable to *in vivo* conditions. The most widely used cell model is represented by the Caco-2 with applications in the study of active transport of mineral nutrients (84, 86).

**FIGURE 4:**
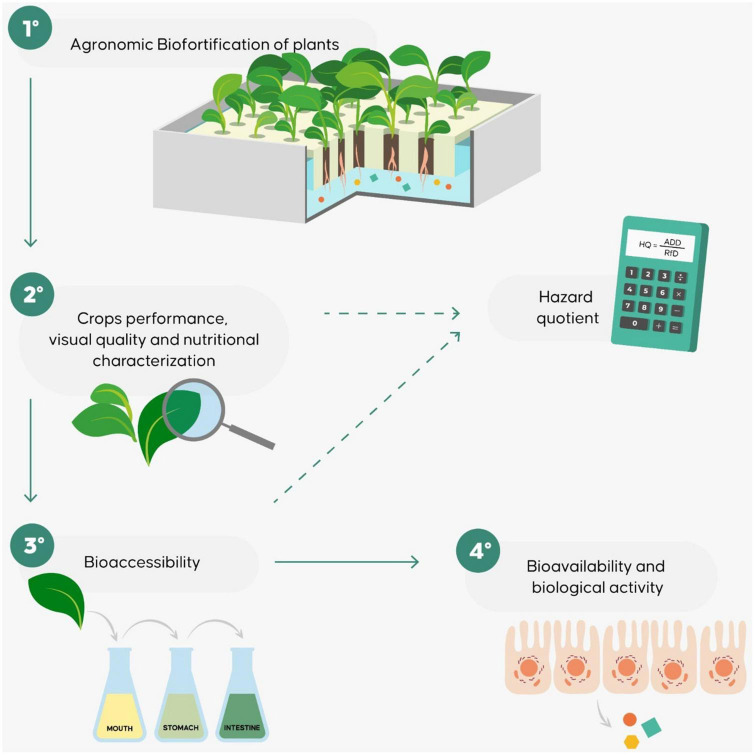
The workflow proposed to evaluate the agronomic, nutritional, and bioefficiency of biofortified products.

Only a few studies, aiming to evaluate biofortified vegetables have been conducted to assess bioaccessibility and bioavailability as part of the biofortification process. In general, these studies used a multidisciplinary workflow based on an evaluation of the efficiency of the biofortification process from a nutritional point of view, taking into consideration bioaccessibility, bioavailability and biological activity as well as agronomic efficiency (12, 80, 84, 86, 87). The approach is summarized in Figure 4. Bioavailability and bioaccessibility assessment provides nutritional and biological information regarding biofortified products generally not furnished by agronomic biofortification studies. In addition, it can be used to improve the development of biofortified foods with regard to the choice of the species or of the type of food processing to use to produce plant foods that satisfy consumer needs (76, 80, 86, 88).

## Ongoing trends and the challenges ahead

In addition to the three specific categories of individuals discussed above, other groups may be interested in switching over to the consumption of soilless produced vegetables containing higher quantities of essential micronutrients/compounds and low concentrations of undesired elements or compounds.

Nickel (Ni) is an ubiquitous trace element and the commonest cause of metal allergy. Individuals sensitized to Ni through dermal contact and who have allergic contact dermatitis (some have estimated to that it affects up to 15% of women, but it is an undiagnosed entity) develop hand eczema from oral, as well as dermal, exposure to Ni salts. Oral intakes of Ni as low as approximately 500 μg per day have been reported to aggravate hand eczema in Ni sensitized subjects (89). Nickel in soil and water is taken up by living organisms, plants and animals that are food sources for humans; it is therefore present in most of the constituents of a normal diet. The Ni content in fruits and vegetables is on average fourfold higher with respect to food of animal origin (90). At the same time, Ni content in vegetable food products can vary widely, depending on the Ni content in both soil (ranging between 5 and 500 μg g^–1^) and water (between 5 and 100 μg L^–1^). For these reasons, the Ni content in individual foods appears to vary widely depending on a number of variables (90). But independently of the Ni content of the soil, some vegetable products, such as legumes, whole wheat, and cocoa and derivates are known to have a high Ni content. With regard to other types of vegetable food products it is difficult to define what is “high in nickel” because different thresholds have been used by many authors and institutions. In fact, the threshold can range from 0.5 to 0.03 mg kg^–1^. Using the latter threshold, a host of vegetable products including tomatoes and carrots should be considered high in Ni (90). More reliable data about the Ni content in foods are therefore needed. Currently, some hydroponic companies are producing certified vegetables that comply with the requirements of the “Product Technical Specification” regulating the vegetable supply chain and guaranteeing that nickel is absent in food products (91, 92). For the time being there are no studies in the literature regarding the soilless production of vegetables with undetected (verified analytically) nickel. Future studies should thus aim to develop soilless vegetable production methods experimenting with the growing media, irrigation water, fertilizers and cultivars in order to reduce the uptake of Ni and restrict its translocation to the edible parts of the plant and therefore to produce practically Ni-free vegetables for individuals sensitized to Ni.

Nitrate (NO_3_), which is a naturally occurring form of nitrogen, is an integral part of the nitrogen cycle in the environment. Approximately 80% of the nitrates in the daily diet come from the consumption of vegetables, mainly through green-leaf vegetables (93). For the most part NO_3_-accumulating vegetables belong to the *Brassicaceae* (rocket, radish, mustard), *Chenopodiaceae* (beetroot, Swiss chard, spinach) and *Amarantaceae* families. The *Asteraceae* (lettuce) and *Apiaceae* (celery, parsley) include species that are characterized by a high content of NO_3_ (94). Nitrate *per se* is relatively non-toxic; nevertheless once ingested NO_3_ is converted to nitric oxide. It can react with hemoglobin (oxyHb) to form methaemoglobin (metHb), which may impair oxygen delivery to human tissue causing methaemoglobinaemia, or blue baby syndrome. Infants are more susceptible to a syndrome characterized by clinical symptoms such as the blue discoloration of the skin due to the presence of deoxygenated blood and asphyxia. This occurs because young infants have less of the reductase needed to reconvert the metHb back to oxyHb and have low NO_3_-reducing activity due to low gastric acidity (94). Given these considerations, the European Food Safety Authority (EFSA) released a statement on possible public health risks for infants and young children linked to the presence of nitrates in leafy vegetables, and established that the maximum NO_3_ concentration for baby foods is 200 mg kg^–1^ fresh weight, including vegetables (95). Some of the strategies that can be used to reduce the NO_3_ content in vegetables grown using hydroponic systems are: (i) removing part or all of the nitrate nitrogen from the nutrient solution a few days before harvesting; (ii) using nutrient solutions with NO_3_-N and NH_4_-N rather than nitrate nitrogen only (96); (iii) growing vegetables under high light intensity conditions (97).

Beyond the possible acute health effects of nitrates in infants and young children who consume spinach and lettuce, it should be remembered that NO_3_ supplementation enhances nitric oxide (NO) bioavailability *via* the NO_3_–nitrite–NO pathway, which is involved in several physiological processes that could potentially improve skeletal muscle function. In fact, there is evidence that dietary NO_3_ supplementation has ergogenic effects during endurance and sprint-type exercises and others types of physical activities such as weightlifting (93, 98). The limited number of studies as well as the diversity of the results published impede us from drawing any clear conclusions about NO_3_ supplementation in athletes. As far as endurance sports are concerned, the dose necessary for a significant effect continues to be unclear since in some trials acute doses of 12 mmol of NO_3_ were used while in others smaller doses (up to 6 mmol day^–1^ of NO_3_) were utilized (93). Despite the differences in the supplementation dosage (from 32.5 mg NO_3_ to 6.4 mmol of NO_3_) used in the trials and in the periods of supplementation (from acute to chronic over 6 days), the limited data existing in the literature suggest that dietary nitrate supplementation could potentially enhance weightlifting performance (98). Further research should attempt to analyze the ergogenic effect of nitrate supplementation on athletes as well as to study the optimal sources and the most suitable species and doses of nitrates and the best hydroponic systems to produce nitrate-enriched tailored vegetables. The challenges ahead include those of identifying the categories of people who would most benefit from vegetable biofortification and determining the sustainability of the production processes. According to the “*Farm to Fork*” strategy, which is at the heart of the European Green Deal, food systems cannot be resilient to crises if they are not sustainable. Our food systems need therefore to be set on a sustainable path which will also create new opportunities for operators in the food value chain (99). While it may seem counterintuitive, high−tech soilless cultivation systems and organic agriculture have several converging points in view of a sustainable use of the planet’s natural resources (100). In the future those working in the sector should aim to verify that soilless cultivation systems have a low environmental impact and that biofortified vegetables are high quality products.

## Conclusion

Enhancing the precision biofortification processes through soilless systems and understanding the aspects related to bioaccessibility and bioavailability of a particular element from a biofortified vegetables are launching horticultural science into the era of personalized nutrition. Consequently, it is clear that the multidisciplinary approach toward tailored foods is a winning one and must increasingly include a synergy between agronomic, biological and medical skills. Therefore, for further goals vegetable biofortification trials could be joined to clinical studies for assessing the potential additional benefits of the emerging biofortified vegetables for specific categories of people.

## Author contributions

MR, MD’I, and FS contributed to the study concept and design of the manuscript, tables and figure preparation, and edition. MR, MD’I, FS, and SM critically reviewed the article. MR and MD’I contributed to the acquisition and analysis of data and drafted the manuscript. All authors gave final approval for all aspects of the work, agreed to be fully accountable for ensuring the integrity and accuracy of the work, and read and approved the final manuscript.
